# Coronary Artery Anomaly With Absent Common Iliac Artery and Genitourinary Malformation: A Rare Case Report

**DOI:** 10.7759/cureus.81331

**Published:** 2025-03-28

**Authors:** Idriss Souko, Motaz Y Majthoob, Dogan Emre Sert, Imthiaz Manoly, Obaid Aljassim

**Affiliations:** 1 Cardiothoracic Surgery, Dubai Hospital, Dubai, ARE

**Keywords:** absent common iliac artery, cabg surgery, coronary artery anomalies, ecls, genitourinary malformation

## Abstract

Congenital coronary artery anomalies are rare, and while often asymptomatic, some anomalies can pose life-threatening risks. Peripheral vascular anomalies, particularly involving the iliac arteries, are even rarer and may go undetected unless complications arise. Their presence can significantly impact interventional and surgical strategies, particularly when extracorporeal circulatory support is required during cardiac surgery.

We report a rare case of a 37-year-old male presenting with acute coronary syndrome and an anomalous left circumflex coronary artery originating from the right coronary sinus. Coronary angiography revealed multivessel disease, and the patient underwent coronary artery bypass graft surgery. Preoperative imaging revealed the absence of the right common iliac artery, with the distal right external iliac artery reconstituted by collateral circulation. Additionally, an ectopic, malrotated left kidney was identified. The postoperative course was uneventful, and the patient was discharged on the seventh postoperative day.

This case highlights the importance of suspecting other congenital vascular anomalies in patients with congenital coronary artery anomalies, as these anomalies can pose significant challenges, particularly when mechanical circulatory support is required after coronary artery bypass graft surgery. A comprehensive preoperative imaging workup, including Doppler sonography and thoracoabdominal and pelvic CT, is crucial for detecting such anomalies early, ensuring optimal surgical planning, and avoiding intraoperative surprises.

## Introduction

Congenital coronary artery anomalies are rare, affecting approximately 1% of the general population. There are different varieties of coronary artery anomalies; however, the most common anomaly is the anomalous origin of the left circumflex coronary artery (LCx), either from the right coronary sinus or directly from the right coronary artery (RCA) [1]. In normal anatomy, the left circumflex artery originates as one of the branches of the left main coronary artery. It runs in the atrioventricular sulcus and gives obtuse marginal branches, and sometimes the posterior descending artery [2].

Coronary artery anomalies are often detected incidentally during coronary angiography (CAG) [3]. While typically asymptomatic, certain coronary anomalies can pose life-threatening risks, particularly in young athletes, where sudden cardiac death may be the first manifestation [4].

Vascular malformations of the iliac and femoral vessels are much rarer than those involving the thoracic and abdominal aorta. They are often detected incidentally or during the evaluation of lower extremity ischemia. The exact prevalence of iliofemoral anomalies remains uncertain, with only a few cases identified in large angiographic studies of symptomatic patients [5]. These anomalies are usually associated with genitourinary abnormalities, as shown in some case reports [6-9]. 

The relationship between coronary artery anomalies and peripheral vascular anomalies remains unclear. However, in cases of post-cardiotomy cardiogenic shock following coronary artery bypass graft (CABG) surgery, the use of extracorporeal life support (ECLS) devices, such as an intra-aortic balloon pump (IABP) or extracorporeal membrane oxygenation (ECMO), may become necessary. The presence of peripheral vascular anomalies, especially in the iliofemoral region, can hinder the insertion of these devices. This underscores the critical importance of a thorough preoperative evaluation for such anomalies in patients with coronary artery disease, particularly those with coronary anomalies, prior to CABG surgery.

We present a rare case of concurrent coronary and peripheral vascular anomalies in a patient with acute coronary syndrome (ACS) who underwent CABG surgery.

## Case presentation

A 37-year-old male patient with a history of diabetes and dyslipidemia presented to the emergency department with acute retrosternal chest pain. He was vitally stable, with a blood pressure of 115/85 mmHg, heart rate of 91/min, respiratory rate of 18/min, and peripheral oxygen saturation of 95%. Laboratory tests revealed elevated serum troponin, creatine kinase-MB (CK-MB), dyslipidemia, and hyperglycemia (Table 1).

**Table 1:** Laboratory investigations at hospital admission CKMB: creatine kinase-MB, LDL: low-density lipoprotein, Non-HDL: non-high-density lipoprotein, HbA1C: hemoglobin A1c

Laboratory parameters	Results	Reference value
Troponin T	4087 ng/L	<14 ng/L
CKMB	20.4 ng/mL	<6.23 ng/mL
Total cholesterol fasting	275 mg/dL	<190 mg/dL
Triglycerides	131 mg/dL	<150 mg/dL
LDL-cholesterol	205 mg/dL	<115 mg/dL
Non-HDL cholesterol	231 mg/dL	<145 mg/dL
Random blood glucose	159 mg/dL	65 - 140 mg/dL
HbA1C	6.5%	<5.7 %

**Figure 1:**
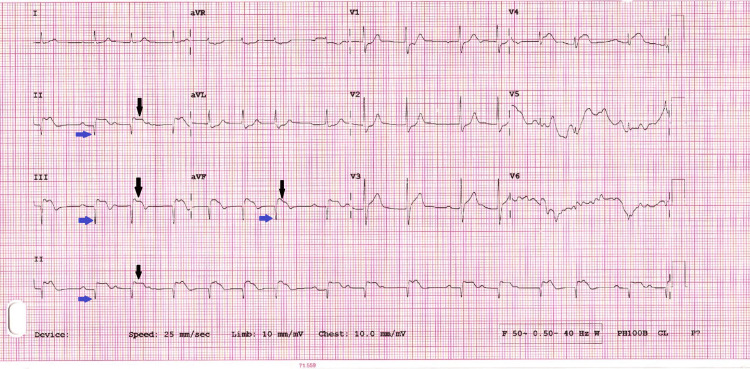
ECG on admission showing ST elevation (black arrows) and Q waves (blue arrows) in leads II, III, and aVF indicating inferior myocardial infarction ECG: electrocardiogram

The electrocardiogram (Figure 1) showed signs of an inferior myocardial infarction. Transthoracic echocardiography (TTE) revealed moderate to severe impairment of left ventricular systolic function, with an ejection fraction (EF) between 30% and 35%. With a suspicion of ACS, the patient was promptly taken for CAG.

**Figure 2:**
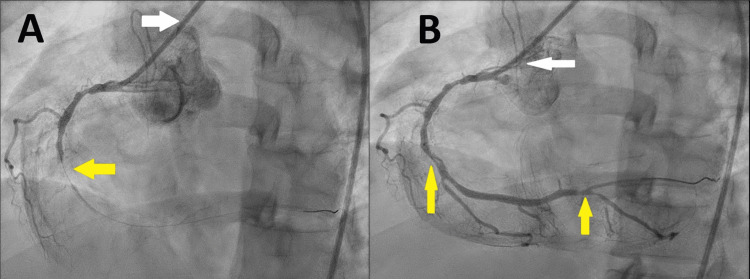
Selective CAG of the right coronary artery A: CAG catheter (white arrow) inserted into the RCA ostium, showing an occluded RCA in its midsegment (yellow arrow). B: CAG catheter (white arrow) inserted into the right RCA ostium, showing restored flow to the RCA with significant lesions in its mid- and distal segments (yellow arrows) CAG: coronary angiography; RCA: right coronary artery

Initially, right femoral artery access was attempted, but advancing the guidewire was unsuccessful. The decision was made to switch to the left femoral artery, which was successful. CAG revealed an occluded RCA with a thrombus, identified as the culprit lesion (Figure 2A).

**Figure 3:**
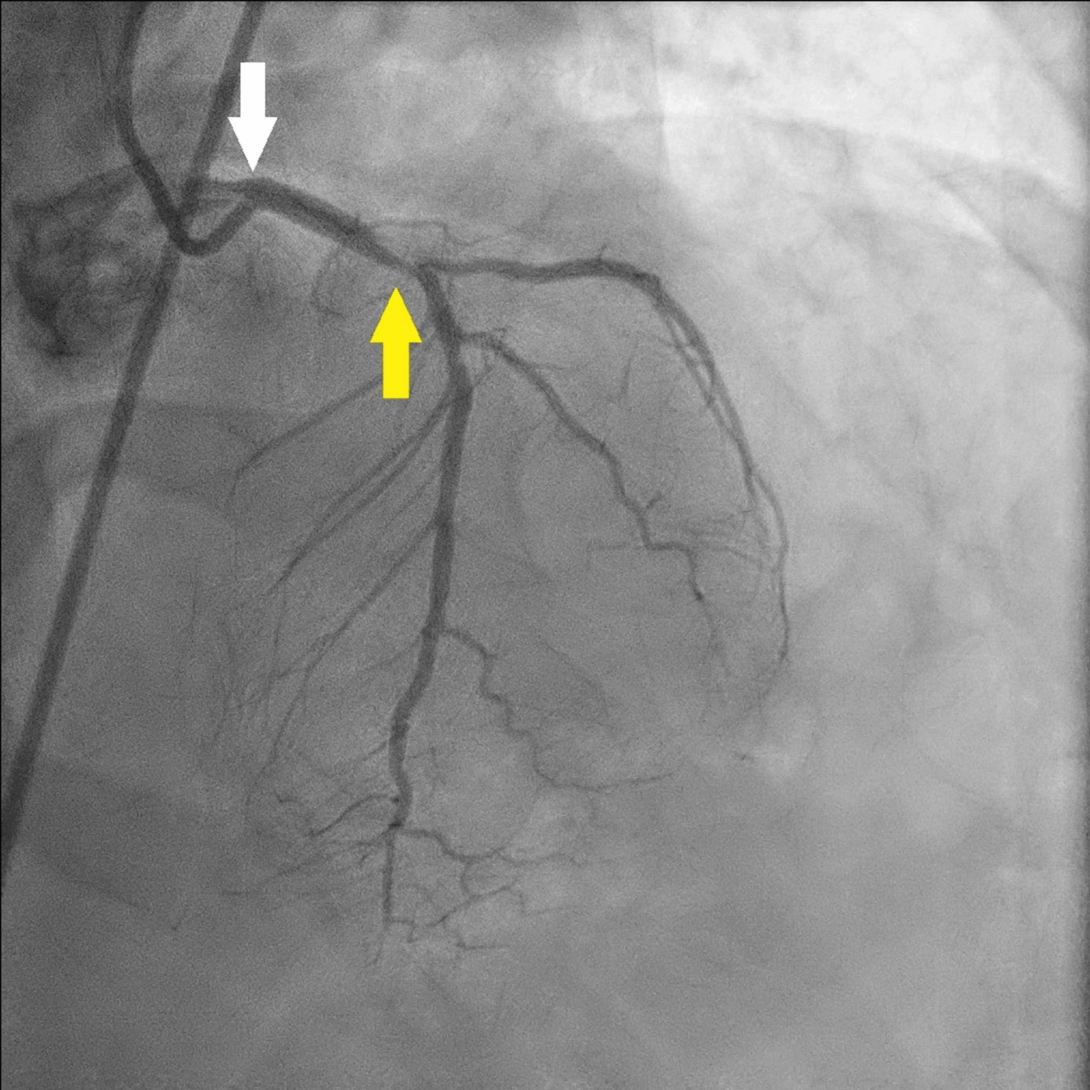
Selective CAG (white arrow) of the LAD showing a significant lesion in its proximal segment (yellow arrow) CAG: coronary angiography; LAD: left anterior descending artery

A significant lesion was also noted in the proximal left anterior descending artery (LAD) (Figure 3).

**Figure 4:**
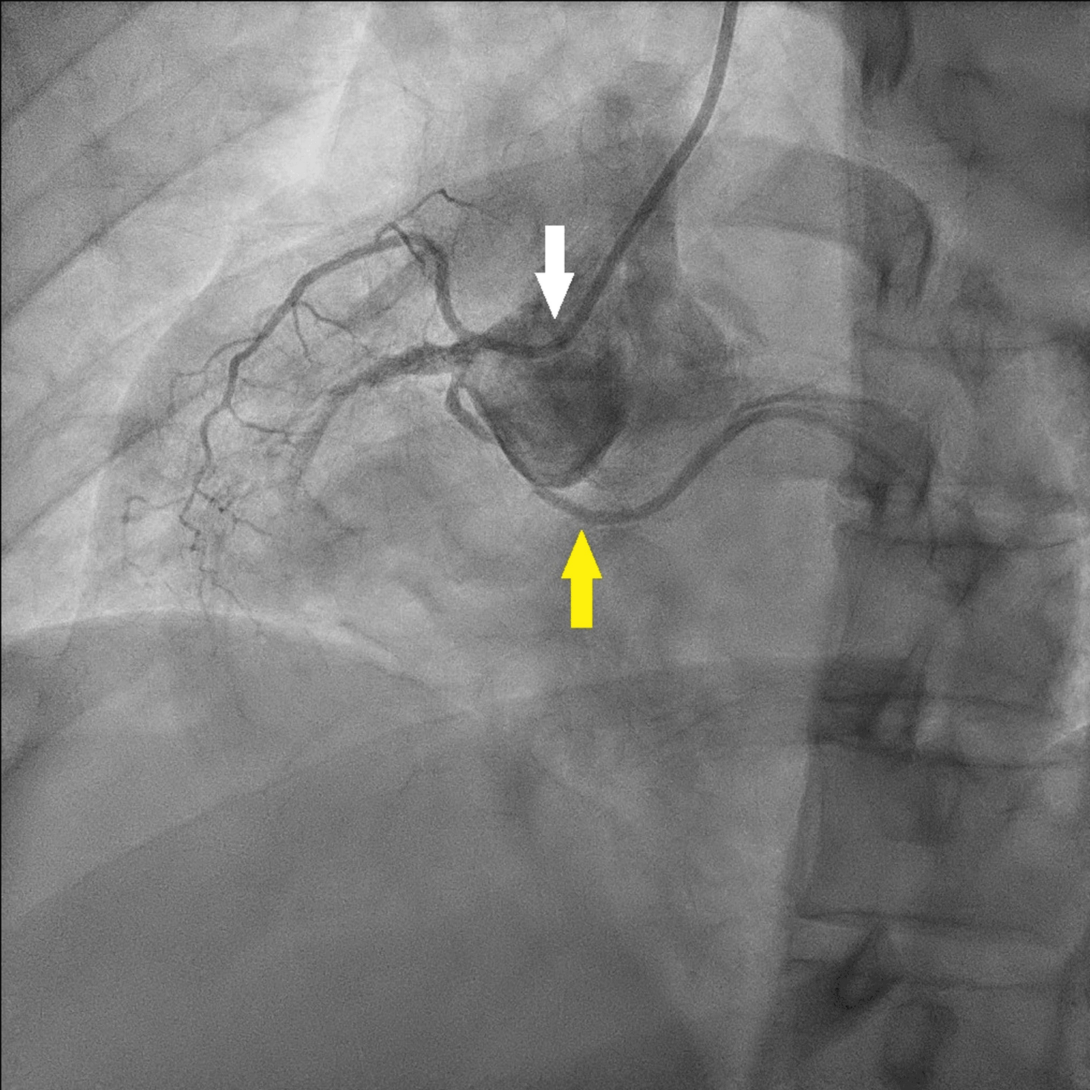
CAG catheter in the right coronary sinus (white arrow) showing abnormal origin of the LCx artery from the right coronary sinus (yellow arrow) CAG: coronary angiography; LCx: left circumflex artery

The left circumflex (LCx) artery originated from the right coronary sinus and showed a significant long-segment lesion (Figure 4).

Balloon dilatation percutaneous coronary intervention successfully restored flow in the occluded RCA (Figure 2B). The distal LCx branches were small, and the coronary circulation was right-dominant.

After receiving a bolus dose of 25 mcg/kg of Tirofiban (Aggrasta) via intravenous infusion, the patient was transferred to the coronary care unit and placed on an Aggrastat infusion (0.15 mcg/kg/min) for stabilization and close monitoring. Over the following days, he remained free of angina and hemodynamically stable.

Given the presence of multivessel disease, the heart team recommended CABG surgery during his inpatient stay.

**Figure 5:**
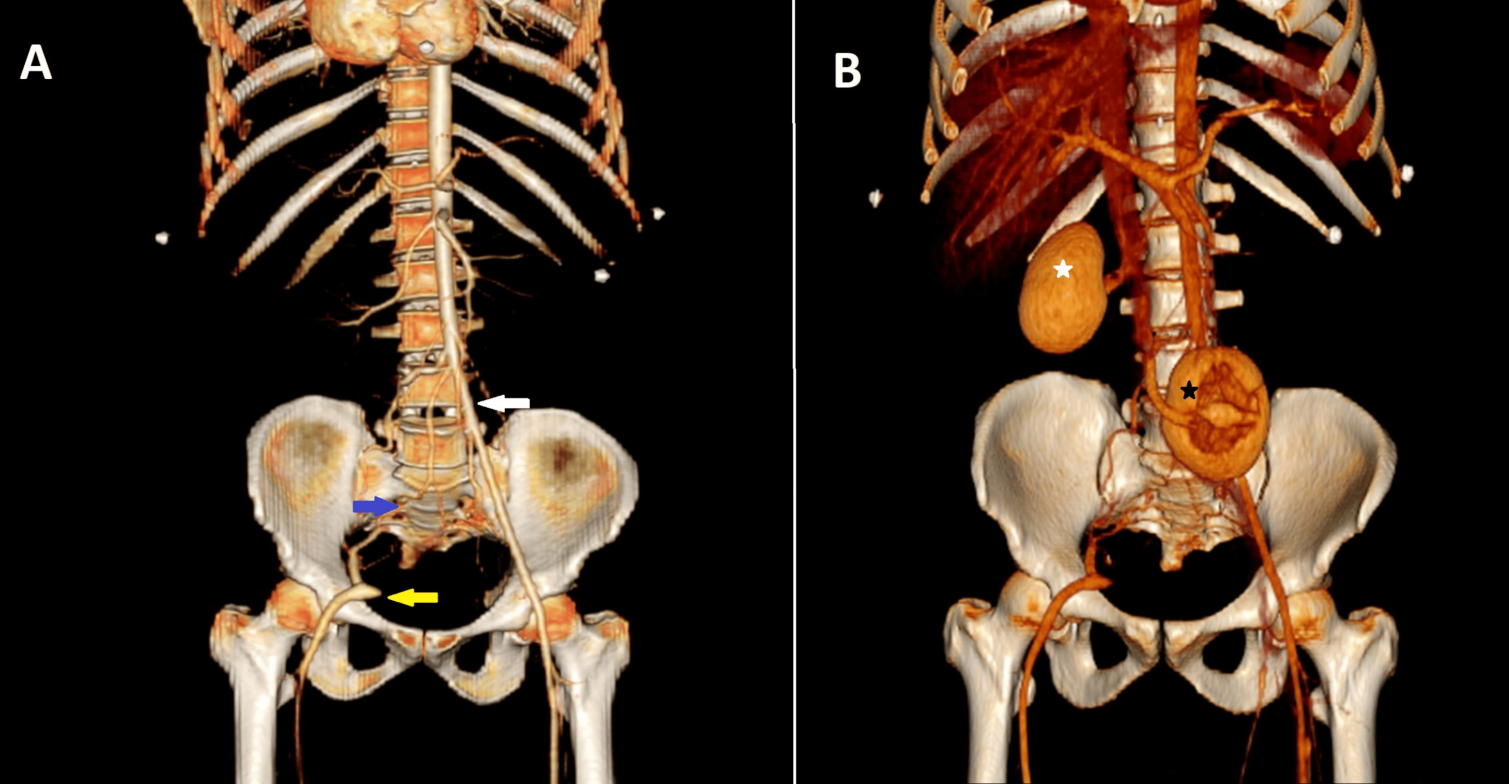
Frontal, three-dimensional (3D) volume-rendered image of the abdomen and pelvis, A: normal left common iliac artery (white arrow) and absent right common iliac artery. The right external iliac artery (yellow arrow) is reconstituted via multiple lumbosacral collaterals (blue arrow). B: The right kidney is in normal position (white star), and the left kidney is malrotated and positioned in the pelvis (black star)

During hospitalization, the patient developed a hematoma in the right thigh, raising suspicion of a vascular injury from the guidewire attempt. A computed tomography angiography of the abdomen and pelvis ruled out any vascular injury. However, it revealed a normal left common iliac artery with well-developed internal and external branches. In contrast, the right common iliac artery was absent, with the aorta terminating abruptly at the expected origin of the right common iliac artery. The proximal portion of the right external iliac artery was not visualized; however, its distal portion was reconstituted via multiple lumbosacral collaterals (Figure 5A).

Further imaging revealed that the right kidney was in its normal position with a normal vascular supply. However, the left kidney was ectopic, malrotated (with the renal pelvis facing anteriorly), and located in the pelvis (Figure 5B).

**Figure 6:**
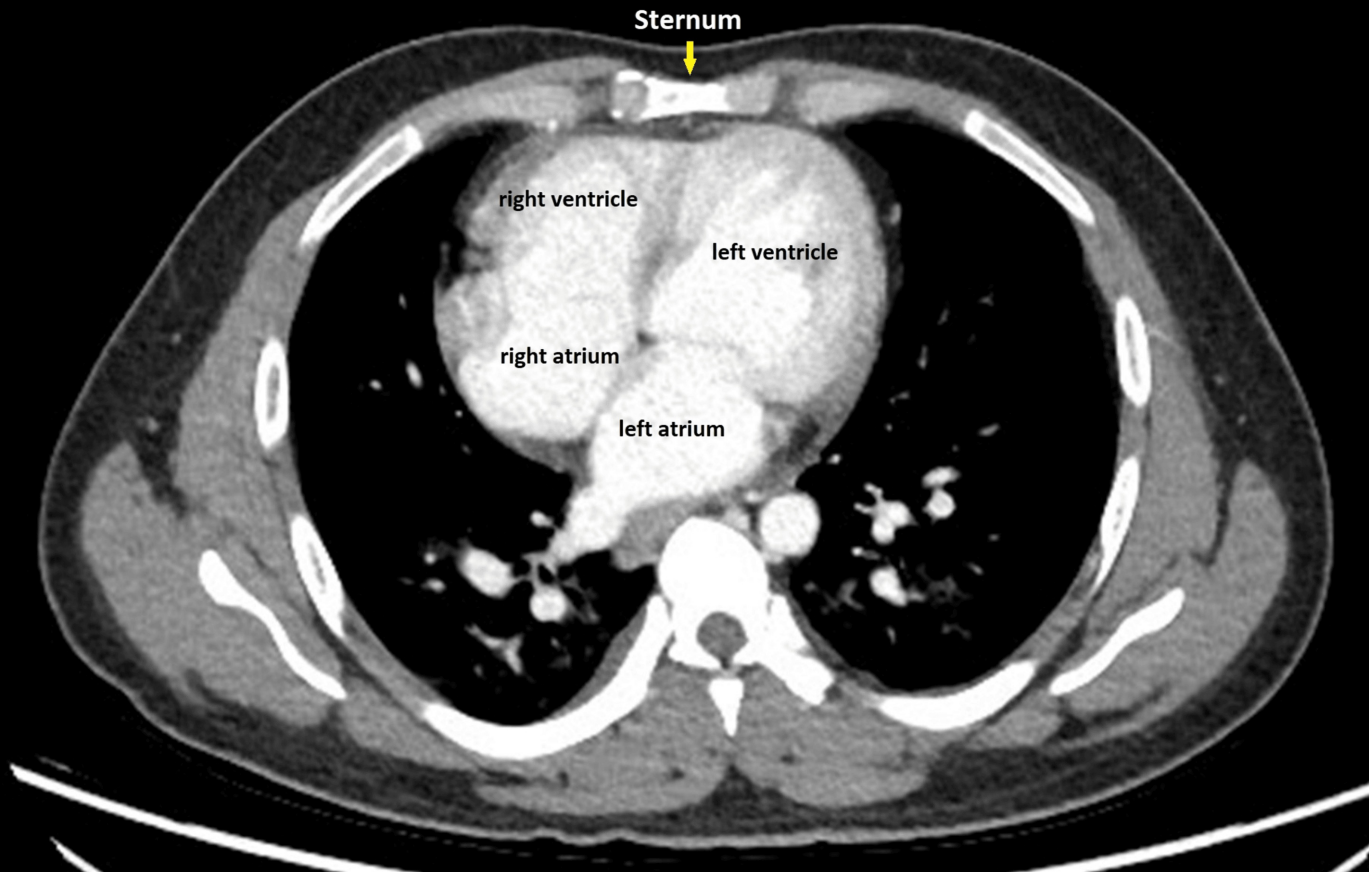
Axial CT scan of the chest showing an anticlockwise rotated heart CT: computed tomography

In addition to the coronary and iliac artery anomalies, computed tomography (CT) imaging of the chest revealed anticlockwise rotation of the heart. This resulted in the right ventricle being positioned further right to the sternum, while the left ventricle assumed a more substernal location (Figure 6).

In normal anatomy, the right ventricle is typically located in a substernal position, while the left ventricle is positioned more laterally and posteriorly.

On physical examination, the patient was noted to be short-statured (160 cm, 57 kg) with short extremities. Genitourinary examination revealed hypospadias.

CABG surgery was performed one week after admission using a heart-lung machine with aortic and right atrial cannulation. The cardiopulmonary bypass time was 158 minutes, and the aortic cross-clamp time was 96 minutes. The left internal thoracic artery (LIMA) was grafted to the LAD, and the radial artery was used for grafting the distal RCA territory. As anticipated, the LCx branches were small (<1 mm in diameter). Given the right-dominant coronary circulation and the limited size of the LCx branches, grafting to the LCx branches was abandoned.

Positioning of the heart for exposure of the coronary arteries was extremely difficult due to the malpositioned heart.

The postoperative course was uneventful. The right thigh hematoma had subsided, and the patient was discharged on the seventh postoperative day. On follow-up in our outpatient clinic at 2 and 6 weeks after discharge, he was generally stable. 

## Discussion

Congenital anomalies of the coronary arteries are rare and affect 1% of the general population. The anomalous origin of the left circumflex coronary artery (LCx) from the right coronary sinus or the right coronary artery (RCA) is one of the most common congenital coronary anomalies observed in patients undergoing CAG [1]. Most individuals with these anomalies are asymptomatic and are diagnosed incidentally during CAG [3].

Certain coronary anomalies can be life-threatening, particularly in young, seemingly healthy athletes. However, the precise causes and prevalence of sudden death associated with these anomalies remain uncertain. In many cases, sudden death is both the first and only symptom. Pathological studies have shown that among patients with an anomalous left coronary artery originating from the right sinus, 59% died before reaching the age of 20, primarily during or shortly after intense physical activity [4].

In acute coronary syndrome, CAG is essential and often performed via femoral artery access due to its longer history of use and technical simplicity compared to the radial artery approach [10]. Difficulty in introducing the CAG guidewire is typically attributed to atherosclerotic lesions of the iliofemoral territory [11]. However, in extremely rare cases, congenital vascular abnormalities, such as the absence of iliac arteries, should be considered. These anomalies are often asymptomatic due to collateral formation, which provides enough blood supply to the lower extremities [12].

The majority of these anomalies are unilateral, with a predominant involvement of the right side [6]. Additionally, they are often associated with genitourinary anomalies [6,7], as observed in our case (Figure 5B).

The association between coronary artery anomalies and the absence of the iliac arteries remains unclear. However, in the context of coronary artery bypass graft (CABG) surgery, as in our case, the presence of peripheral vascular anomalies presents unique challenges, particularly when weaning from cardiopulmonary bypass proves difficult. In such situations, ECLS systems, including IABP or ECMO, may be required to support cardiac function. However, in patients with an absent iliac artery, the application of these peripheral support devices becomes impossible.

To mitigate these challenges, preoperative thoracoabdominal and pelvic CT imaging is recommended for patients with known coronary anomalies. Such imaging facilitates the early detection of peripheral vascular anomalies, allowing for proper surgical planning and ensuring alternative strategies for circulatory support if ECMO or IABP use becomes necessary. Furthermore, in patients with coronary anomalies, identifying these peripheral vascular anomalies preoperatively may also aid in predicting and managing future complications, such as limb hypoperfusion symptoms, which can arise due to underlying congenital vascular abnormalities.

The absence of the common iliac artery is frequently associated with genitourinary anomalies, including ectopic kidneys and hypospadias [6,7], as observed in our case. If kidney transplantation is needed in the future, this vascular anomaly poses a significant challenge, as implanting the donor kidney on the side of the absent iliac vessel may be technically difficult.

## Conclusions

This case highlights the need to suspect iliofemoral vascular anomalies in patients with congenital coronary artery anomalies. The absence of an iliac artery can hinder the use of peripheral circulatory support devices like IABP or ECMO, posing challenges in cardiac surgery. Preoperative imaging, including thoracoabdominal and pelvic CT, is crucial to detect these anomalies early, allowing for better surgical planning and avoiding intraoperative surprises.
